# Synthesis and Characterization of Hematite-Based Nanocomposites as Promising Catalysts for Indigo Carmine Oxidation

**DOI:** 10.3390/nano12142511

**Published:** 2022-07-21

**Authors:** Andrei Cristian Kuncser, Arpad Mihai Rostas, Rodica Zavoianu, Octavian Dumitru Pavel, Ioana Dorina Vlaicu, Mihaela Badea, Daniela Cristina Culita, Alina Tirsoaga, Rodica Olar

**Affiliations:** 1 Laboratory of Atomic Structures and Defects in Advanced Materials, National Institute of Materials Physics, 405A Atomiştilor Str., Ilfov, 077125 Măgurele, Romania; andrei.kuncser@infim.ro (A.C.K.); arpad.rostas@infim.ro (A.M.R.); ioana.vlaicu@infim.ro (I.D.V.); 2 Department of Organic Chemistry, Biochemistry and Catalysis, Faculty of Chemistry, University of Bucharest, 4-12 Regina Elisabeta Av., S3, 030018 Bucharest, Romania; rodica.zavoianu@chimie.unibuc.ro; 3 Department of Inorganic Chemistry, Faculty of Chemistry, University of Bucharest, 90-92 Panduri Str., S5, 050663 Bucharest, Romania; mihaela.badea@chimie.unibuc.ro; 4 Ilie Murgulescu Institute of Physical Chemistry, 202 Splaiul Independentei, 060021 Bucharest, Romania; dculita@icf.ro; 5 Department of Physical Chemistry, Faculty of Chemistry, University of Bucharest, 4-12 Regina Elisabeta Av., S3, 030018 Bucharest, Romania; alina.tirsoaga@chimie.unibuc.ro

**Keywords:** hematite, nanocomposite, morphology, catalysis, indigo carmine

## Abstract

The hematite-based nanomaterials are involved in several catalytic organic and inorganic processes, including water decontamination from organic pollutants. In order to develop such species, a series of bimetallic hematite-based nanocomposites were obtained by some goethite composites-controlled calcination. Their composition consists of various phases such as α-FeOOH, α-Fe_2_O_3_ or γ-Fe_2_O_3_ combined with amorphous (Mn_2_O_3_, Co_3_O_4_, NiO, ZnO) or crystalline (CuO) oxides of the second transition ion from the structure. The component dimensions, either in the 10–30 or in the 100–200 nm range, together with the quasi-spherical or nanorod-like shapes, were provided by Mössbauer spectroscopy and powder X-ray diffraction as well as transmission electron microscopy data. The textural characterization showed a decrease in the specific area of the hematite-based nanocomposites compared with corresponding goethites, with the pore volume ranging between 0.219 and 0.278 cm^3^g^−1^. The best catalytic activity concerning indigo carmine removal from water in hydrogen peroxide presence was exhibited by a copper-containing hematite-based nanocomposite sample that reached a dye removal extent of over 99%, which correlates with both the base/acid site ratio and pore size. Moreover, **Cu-hbnc** preserves its catalytic activity even after four recyclings, when it still reached a dye removal extent higher than 90%.

## 1. Introduction

Iron oxides such as α-Fe_2_O_3_ (hematite), γ-Fe_2_O_3_ (maghemite), and Fe_3_O_4_ (magnetite) are some of the most important transition-metal oxides with essential industrial importance. Among these, hematite is the most stable species in an aqueous medium at ambient conditions and over a wide pH range. Its earth-abundance, low cost, and environmentally friendly behavior make it a species frequently used as a catalyst for both inorganic and organic processes, alone or in bi- or trimetallic composites [1].

From this point of view, many studies report data concerning the hematite-based (HB) catalyst’s abilities to eliminate organic and inorganic pollutants from water. Hence, the HB systems are used alone or with oxidants such as ozone, hydrogen peroxide, or persulfate for organic pollutants elimination in water decontamination and disinfection processes [2,3,4,5,6,7,8,9,10,11,12,13,14,15,16,17,18,19,20]. Good results were obtained for eliminating various organic drugs such as ciprofloxacin [2], acetaminophen [3], diclofenac [4], and sulfasalazine [5] from wastewaters. Moreover, a wide range of recalcitrant organic chemical pollutants such as benzene [6], toluene [7], phenols [6,8], rhodamine B [9,10,11], methylene blue [11,12], and bromophenol blue dyes [13] was also efficiently removed from wastewater using such catalysts.

In addition, significant efforts have been focused on improving the catalytic activity of HB catalysts by introducing a second transition ion into its composition. As a result, there are several studies concerning hematite-based nanocomposites that have hydroxide/oxide of Fe(III), Mn(II,III), Co(II,III), Ni(II), Cu(II), or Zn(II) in their structure.

Among these, the systems with MnO_2_ or Mn_2_O_3_ are valuable in seawater splitting [14] or water decontamination [15,16], while those containing Co_3_O_4_ perform in water pollutants degradation in the presence of H_2_O_2_ or persulfate [17]. Moreover, a bimetallic material with NiO has been developed as a catalyst for water depollution [18]; a system bearing CuO showed valuable properties in the degradation of methylene blue [19], while systems containing ZnO were developed as photocatalysts for environmental remediation [20].

Usually, hematite is mainly obtained by the wet-chemical precipitation of FeO(OH) species followed by controlled pyrolysis [21]. Considering the bimetallic oxide composites, these are obtained through the same calcination method after the hydroxide/oxyhydroxide coprecipitation in a proper molar ratio with [21] or without the help of a template [15,16].

Recently, we reported the synthesis of a series of goethite-based composites by a soft synthesis method. The Mn(III)-containing species in this series exhibited promising catalytic activity in cyclooctene oxidation [22]. In this work, we extended the study in order to obtain bimetallic hematite-based nanocomposites (**hbnc**) with improved catalytic properties by the controlled calcination of the goethite materials. Furthermore, the influence of the second metallic ion and structural modifications on their catalytic behavior in the oxidative removal of a dye contaminant from wastewater was investigated. The originality of this study consists of the use of materials with complex compositions that are easily synthesized for this purpose.

**Figure 1:**
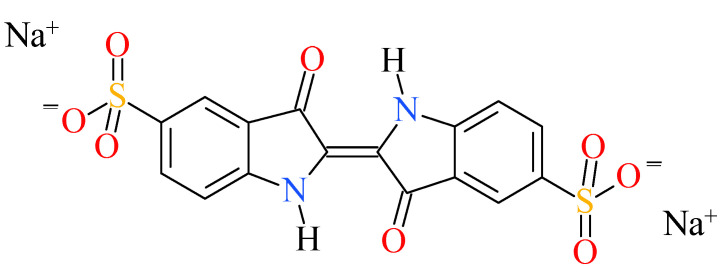
The chemical structure of indigo carmine.

The selected dye contaminant was disodium (2E)-3-oxo-2-(3-oxo-5-sulfonato-2,3-dihydro-1H-indol-2-ylidene)-2,3-dihydro-1H-indole-5-sulfonate (also called indigo carmine (IC)) (Figure 1).

IC is an indigoid water-soluble dye used as synthetic food color with maximum permitted levels between 50 and 500 mg/kg of food [23]. When its concentration exceeds 500 mg/kg body weight/day, it may become toxic, leading to heart failure and potentially carcinogenic risks [24,25]. IC mineralization by photocatalytic advanced oxidation processes (AOP) using TiO_2_ as a photocatalyst was difficult since there was no noticeable loss in the total organic content (TOC) value and release of inorganic ions even if the water coloration disappeared [26]. Recent investigations have shown that MnO_2_ could efficiently remove indigo carmine from polluted water by a combined process using both photo and ultrasonic activation and peroxydisulfate (S_2_O_8_^−^) as an oxidation agent [27]. It was considered that the catalytic activity of MnO_2_ was due to its high affinity for O_2_ and its efficient dye adsorption. However, developing AOP using greener oxidants such as hydrogen peroxide would be of higher interest. Therefore, in this work, we have tested the catalytic activity of the synthesized **hbnc** for the oxidative degradation of IC with H_2_O_2_.

## 2. Materials and Methods

### 2.1. Reagents

The indigo carmine (IC), e.g., disodium (2E)-3-oxo-2-(3-oxo-5-sulfonato-2,3-dihydro-1H-indol-2-ylidene)-2,3-dihydro-1H-indole-5-sulfonate and H_2_O_2_ (30%), were purchased from Sigma-Aldrich (Saint Louis, MO, USA) and Reactivul (Bucharest, Romania). Double distilled water was used for the preparation of simulated wastewater samples.

### 2.2. Instruments and Methods

The Fourier transform infrared (FTIR) spectra were recorded with a Spectrum BX II (Perkin Elmer, USA) spectrometer in the 350–4000 cm^−1^ range by accumulating 32 scans at a resolution of 4 cm^−1^. The powdered samples were diluted into KBr powder in a 1:100 mass ratio, ground thoroughly, and pressed into pellets.

The UV-Vis spectroscopy was performed in solid-state on a V 670 spectrophotometer (Jasco, Easton, MD, USA) with Spectralon as standard in the 200–1500 nm range.

The electron paramagnetic resonance (EPR) spectroscopy measurements were carried out with a Bruker EMX premium X (Bruker, Karlsruhe, Germany) equipped with an X-SHQ 4119HS-W1 X-Band resonator at a microwave frequency of 9.4457 GHz and power of 0.06325 mW. Further measurement parameters were: conversion time 10 ms, time constant 5.12 ms, and modulation amplitude 0.3 mT with one scan. We used a Digital Temperature Control System ER 4131VT with a liquid nitrogen cryostat from Bruker (Bruker, Karlsruhe, Germany) for cooling.

The ^57^Fe Mӧssbauer spectra were obtained in transmission geometry, at 6 K and room temperature, by inserting the samples in a close cycle Janis cryostat (Edina, MN, USA). A SEECO-type spectrometer (Edina, MN, USA) operating under the constant acceleration mode and a ^57^Co(Rh) radioactive source of about 30 mCi activity were used. The acquired ^57^Fe Mӧssbauer spectra were analyzed using the NORMOS software, which allows the decomposition of the measured absorption pattern in spectral components corresponding to different iron nonequivalent positions. In the case of a continuous distribution of the hyperfine parameters, the fitting procedure can be performed using specific routines that provide the envisaged probability distribution function and complementary average hyperfine parameters. The isomer shift is reported relative to the isomer shift of metallic Fe at room temperature.

Powder X-ray diffraction (XRD) patterns were recorded using a Bruker D8 Advance X-ray diffractometer (Bruker, Karlsruhe, Germany) (Cu anode and Ni filter, λ = 1.54184 Å) in Bragg–Brentano configuration. Using the MAUD software, we determined the lattice parameters and the average crystallite size by the Rietveld refinement method. The JEOL 2100 Transmission Electron Microscope (TEM) (Tokyo, Japan), equipped with Energy Dispersive X-Ray (EDS), was used for transmission electron microscopy investigations. Specimens were prepared using the standard powder method.

Nitrogen adsorption–desorption isotherms at 77 K were recorded on a Micromeritics ASAP 2020 automated gas adsorption system (Norcross, GA, USA). The samples were degassed at 200 °C for 6 h under vacuum before analysis. Specific surface areas (S_BET_) were calculated according to the Brunauer-Emmett-Teller (BET) equation, using adsorption data in the relative pressure range between 0.05 and 0.30. The total pore volume (V_total_) was estimated from the amount adsorbed at the relative pressure of 0.99. The pore size distribution (PSD) curves were obtained from the desorption data using the BJH (Barrett–Joyner-Halenda) model.

### 2.3. General Procedure for Hematite-Based Nanocomposites Synthesis

The hematite-based nanocomposites were synthesized by the thermal decomposition of goethite and goethite nanocomposites previously reported [22]. These raw materials were obtained by a soft chemical synthesis consisting of M(II) (M: Co, Ni, Cu, Zn) acetate hydrolysis in a water suspension of goethite.

As for HB-nanocomposites, a mass of 2 g from each sample was poured into a porcelain crucible. The samples were then heated with a rate of 10 °C/min up to 350 °C, kept at this temperature for two hours, and then slowly cooled to room temperature in a muffle furnace with an open-air circulation of type LabTech DAIHAN LabtechCo Ltd. (Daihan, Seoul, South Korea).

### 2.4. Catalytic Tests

This study evaluates the catalytic activities of the synthesized materials in the oxidative degradation of indigo carmine dye from wastewater using hydrogen peroxide as an environmentally friendly oxidation agent.

The catalytic oxidation of IC was performed in a batch system under stirring at 150 rpm at 25 °C using 1 wt% catalyst in simulated wastewater containing different concentrations of IC in the range of 0.015 up to 0.09 mM and H_2_O_2_ (30 wt%) as an oxidation agent at different molar ratios H_2_O_2_/IC in the range 16.3–3624.7. In order to highlight the effect of the catalyst, blank tests were performed without adding the catalyst to the reaction mixture.

Five recycling tests were performed only for the most active catalyst (**Cu-hbnc**) and the unmodified **Fe-hbnc**. To this aim, the catalyst recovered after the first reaction cycle (2 h, 150 rpm, 25 °C, 1 wt% catalyst, 0.03 mM initial concentration of IC and 32.6/1 molar ratio H_2_O_2_/IC) was used in the following reaction cycle using a fresh portion of simulated wastewater.

The dye removal extent (DR%) was determined using UV-Vis spectrometry by monitoring the variation of the absorption maximum at λ = 610 nm specific to IC using double beam UV-Vis spectrometer Jasco V-650. The concentrations of H_2_O_2_ at the end of the catalytic tests were determined by the spectrophotometric methods 209 and 210 developed on Aqualytic spectrophotometer AL 800/SpectroDirect (Dortmund, Germany) using the specific reagent kits for the concentrations range of 0.01–0.5 mg/L and 0.03–1.5 mg/L H_2_O_2_. (see Supplementary Information).

### 2.5. Base Sites Determination

The total number of base sites was determined using the irreversible adsorption of acrylic acid (pKa = 4.2). Samples of dried solids (0.05 g) were contacted for 2 h (duration required for reaching the equilibrium in the liquid–solid system) with 10 mL of 0.01 M solution of acrylic acid in cyclohexane in brown sealed bottles under mild stirring (150 rot/min) at room temperature. It was assumed that the interaction of the solids with atmospheric CO_2_ and water was negligible since the samples were exposed to the atmosphere only during weighing. The concentration of the solution’s remaining acrylic acid after reaching equilibrium was determined by UV-Vis spectrometry at λ_max_ = 225 nm using the Jasco V-650 spectrometer (Tokyo, Japan). Three parallel determinations were performed for each solid sample, and the obtained results were averaged. The amount of acrylic acid (AA) adsorbed was calculated with the formula:

AA_i_ − AA_f_ = AA_ads_

where indexes i and f refer to the initial and final amounts of acrylic acid in the solution, respectively.

The method is inspired by the one used to determine base sites in hydrotalcite-type materials [28]. However, in the case of these solids, we could not use phenol for the separate determination of strong base sites since phenol is known to give colored combinations with iron.

The total concentration of base sites (CB) was calculated with the formula:

CB = AA_ads_/wt. [mmoles AA/gram of sample]

where wt. is the weight of the solid sample.

### 2.6. Acid Sites Determination

The total number of acid sites was determined by pyridine adsorption. The distribution of acid sites, Lewis and Brönsted, respectively, were calculated from the areas of the corresponding peaks in the DRIFT spectra recorded on an FT/IR-4700 Jasco spectrometer (Tokyo, Japan). Samples of dried solids (0.05 g) were contacted with pyridine aliquots (0.2 μL each) and maintained under inert flow at 90 °C for the removal of physisorbed pyridine. The procedure was repeated until the weight of the sample after two consecutive additions of pyridine became constant (it did not vary with more than 0.0001 g). Then, the DRIFT spectrum of the sample with adsorbed pyridine was recorded, considering the DRIFT spectrum of the freshly dried solid as a background. According to literature data [29,30,31], the bands corresponding to pyridine adsorbed on Lewis acid sites appear in the ranges of 1435–1455 and 1570–1615 cm^−1^, while those corresponding to pyridine adsorbed on Brönsted acid sites appear in the range of 1520–1555 and at 1630 cm^−1^.

## 3. Results and Discussion

Recently, we reported the synthesis and characterization of a series of goethite-based composites of type 6FeO(OH)·MnO(OH)·0.5H_2_O, xFeO(OH)·M(OH)_2_·yH_2_O (M: Co, x = 12, y = 3; M: Ni, x = 7, y = 2), and xFeO(OH)·MO·yH_2_O (M: Cu, x = 5.5, y = 3; M: Zn, x = 6, y = 1.5). These nanomaterials were tested as catalysts for cyclooctene oxidation, and Mn-composite exhibited the most promising potential, its activity decreased by 10% after several successive reaction cycles [22].

In order to improve the catalytic activity for the oxidation of persistent dye pollutants, these nano-goethites, together with the goethite raw material, were calcined at 350 °C for two hours. Thus, a new series of hematite-based nanocomposites (**hbnc**) was obtained. Accordingly, with the known composition of goethite-based composites and data provided by powder XRD, these **hbnc** were formulated and abbreviated as:

3(α-Fe_2_O_3_)·FeO(OH) (**Fe-hbnc**)
4(α-Fe_2_O_3_)·4FeO(OH)·Mn_2_O_3_ (**Mn-hbnc**)
15(α-Fe_2_O_3_)·6FeO(OH)·Co_3_O_4_ (**Co-hbnc**)
2.7(α-Fe_2_O_3_)·1.3(γ-Fe_2_O_3_)·NiO (**Ni-hbnc**)
2.3(α-Fe_2_O_3_)·0.5(γ-Fe_2_O_3_)·CuO (**Cu-hbnc**)
3(α-Fe_2_O_3_)·FeO(OH)·ZnO (**Zn-hbnc**)

The samples were characterized as nanocomposites through IR, UV-Vis-NIR, EPR, and Mössbauer spectroscopy. Powder X-ray diffraction and TEM studies provided their morphology and particle dimension. Furthermore, textural parameters were calculated from nitrogen sorption isotherms.

### 3.1. Hematite-Based Nanocomposites Physico-Chemical Characterization

#### 3.1.1. IR Spectral Investigations

Iron oxide hematite adopts a three-dimensional hexagonal structure developed by oxygen atoms packing in which Fe(III) occupies the octahedral holes. The basic units in this network that generate specific bands in the IR spectra consist of Fe(III) ions connected by oxo bridges. In contrast, the oxyhydroxide species presented in some composites generate the characteristic bands arising from hydroxyl group vibrations.

As a result, the characteristic bands observed in the spectra of synthesized hbnc are summarized in Table 1. The goethite dehydration is accompanied by the disappearance of water characteristic bands from the hematite IR spectra, but the α-FeOOH presence is accompanied by the bands around 3400, 1630, and 1120 cm^−1^. The goethite reminiscence is also responsible for the bands at 460 and 405 cm^−1^ assigned to Fe-O stretching vibrations, while the hematite presence is evidenced by new bands around 550 cm^−1^ [32].

#### 3.1.2. UV-Vis–NIR Spectral Investigations

UV-Vis–NIR spectroscopy represents a valuable tool to establish the oxidation state of the second transition ions, except for Zn(II). The Fe-hbnc sample was used for baseline calibration and as a reference to eliminate the Fe(III) interference. In this condition, this method revealed only the d–d bands characteristic for the second transition ion from the material.

Thus, the **Mn-hbnc** sample exhibits a band at 19,600 cm^−1^ assigned to the spin allowed transition ^5^E_g_→^5^T_2g_ in an octahedral stereochemistry of [Mn(III)O_6_] chromophore [33]. The spectrum is modified compared to the goethite precursor due to Mn(OH)_2_ conversion into Mn_2_O_3_ species [34].

The band at 11,430 cm^−1^ for **Co-hbnc** arises from ^4^T_1g_→^4^T_2g_ spin, which allowed the transition of octahedral [Co(II)O_6_] chromophore. In comparison, that at 18,520 cm^−1^ is characteristic for [Co(III)O_6_] chromophore with the same stereochemistry and can be assigned to ^5^T_2g_→^5^E_g_ transition [33]. According to data reported, at 350 °C, the species formed by Co(OH)_2_ oxidative decomposition is the spinel oxide Co_3_O_4_ [35] that contains both Co(II) and Co(III) ions in the network.

The similar chromophore [Ni(II)O_6_] is responsible for the bands at 14,180 and 17,860 cm^−1^ assigned to ^3^A_2g_→^3^T_2g_ and ^3^A_2g_→^3^T_1g_ transitions, respectively, in the spectrum of **Ni-hbnc**. The large and unsymmetrical band located at 15,040 cm^−1^ for the **Cu-hbnc** sample accounts for a square-planar [Cu(II)O_4_] chromophore, and it can be assigned to the d_xy_→d_x2-y2_ transition [33].

#### 3.1.3. Electron Paramagnetic Resonance Spectroscopy Results

**Figure 2:**
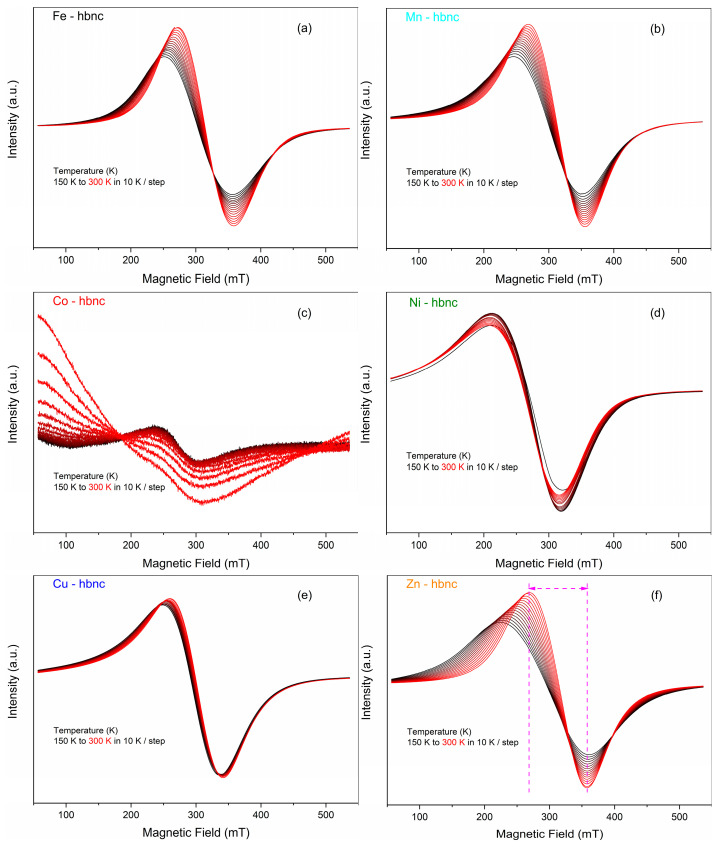
Temperature dependence of the **Fe-hbnc** (**a**), **Mn-hbnc** (**b**), **Co-hbnc** (**c**), **Ni-hbnc** (**d**), **Cu-hbnc** (**e**), and **Zn-hbnc** (**f**) EPR signals.

EPR spectroscopy measurements carried out from low temperature (150 K) to room temperature (300 K) showed broad spectra with a peak-to-peak line width of around 110 mT, specific for Fe_2_O_3_ samples (Figure 2). This broad EPR signal was associated with ferromagnetic exchange interactions between the Fe^3+^ cations [36,37].

**Figure 3:**
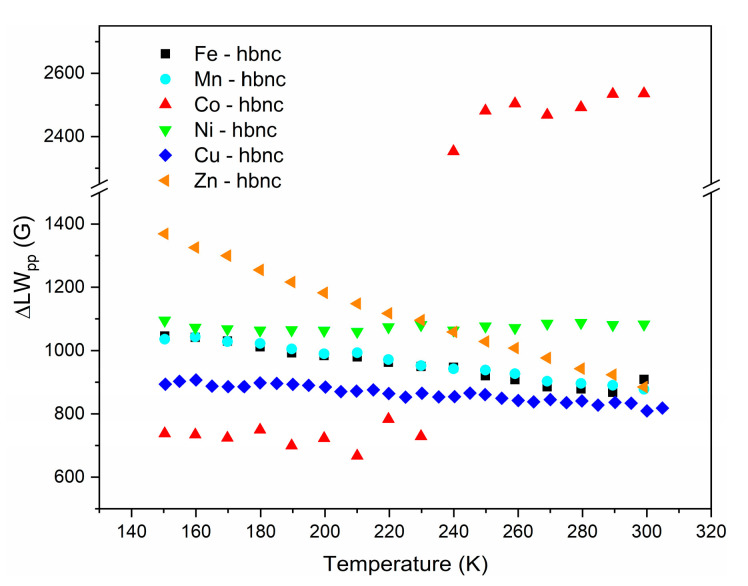
Temperature dependence of the EPR signal peak-to-peak linewidth (ΔLW_PP_) for all species.

Some species such as **Mn-hbnc** and **Ni-hbnc** do not significantly change the Fe_2_O_3_ magnetic properties compared with **Fe-hbnc**. In contrast, these properties were drastically changed for **Co-**, **Zn-**, and **Cu-hbnc** samples. For **Ni-** and **Cu-hbnc** species, the EPR spectra are considerably broadened, which is also evidenced in Figure 3, where the peak-to-peak line width is plotted as a function of the temperature, indicating a strong exchange interaction between the dopant elements and iron. All samples show good doping of the second transition metal ion.

#### 3.1.4. Mössbauer Spectroscopy Results

The room temperature (RT) Mössbauer spectra of the considered samples are presented in Supplementary Figure S1. The Mössbauer parameters (hyperfine magnetic field, isomer shift, quadrupole splitting) obtained from the Mössbauer spectra are shown in Table 2. The fit of these spectra was performed using one or two broad crystalline sextets, an inner sextet considered via a hyperfine field distribution, and, where imposed, a central paramagnetic pattern. The external sextet with the highest hyperfine magnetic field (52 T) was assigned by its specific parameters to the α-Fe_2_O_3_ phase. In contrast, the one with a slightly lower hyperfine magnetic field (48 T) was assigned to γ-Fe_2_O_3_. Finally, the most inner sextet with a field of 32 T was assigned to α-FeOOH, whereas the central paramagnetic pattern to very fine iron oxide nanoparticles was behaving super-paramagnetically at RT. The relative areas of each component provide the relative composition of each phase, and the Mössbauer results in Table 2 are consistent with the Rietveld results in Table 3.

### 3.2. Morpho-Structural Characterization of Samples

#### 3.2.1. Powder X-ray Diffraction Characterization

Rietveld analysis of the XRD spectra was performed on each sample, using one or more of the following phases: α-FeOOH (COD ID 1008766), α-Fe_2_O_3_ (COD ID 2101167), γ-Fe_2_O_3_ (COD ID 1528612), and CuO (COD ID 1526990). In each case, more than one phase was identified (Supplementary Figure S2). In three samples (**Fe**-, **Mn**-, **Co-hbnc**), along with a main α-Fe_2_O_3_ phase, the α-FeOOH was identified. While both **Ni**- and **Cu-hbnc** samples have as main crystal components the α-Fe_2_O_3_ and γ-Fe_2_O_3_, in the case of **Cu-hbnc**, a ternary CuO phase appears as a consequence of copper segregation. **The Zn-hbnc** sample is composed of γ-Fe_2_O_3_ and α-FeOOH phases.

A closer look at the crystal sizes suggests that most samples have two different morphologies, one characterized by nanoparticles (NPs) in the 10–20 nm range and one by NPs in the 100–200 nm range. This behavior is not valid in the **Ni-hbnc** sample, where the size of the nanoentities is relatively uniform in the 30 nm range. It is worth noticing that the crystal sizes in the 100–200 nm domain are greatly affected by errors, while the ones below 30 nm are pretty accurate. Moreover, the assumption for a quasispherical type of NPs under the hood of Rietveld analysis suggests that the morphology associated with NP below 30 nm with accurate size estimation is close to the spherical one.

The lattice parameters obtained on each identified phase do not differ significantly from the reference values (Table 4).

#### 3.2.2. Transmission Electron Microscopy Characterization

**Figure 4:**
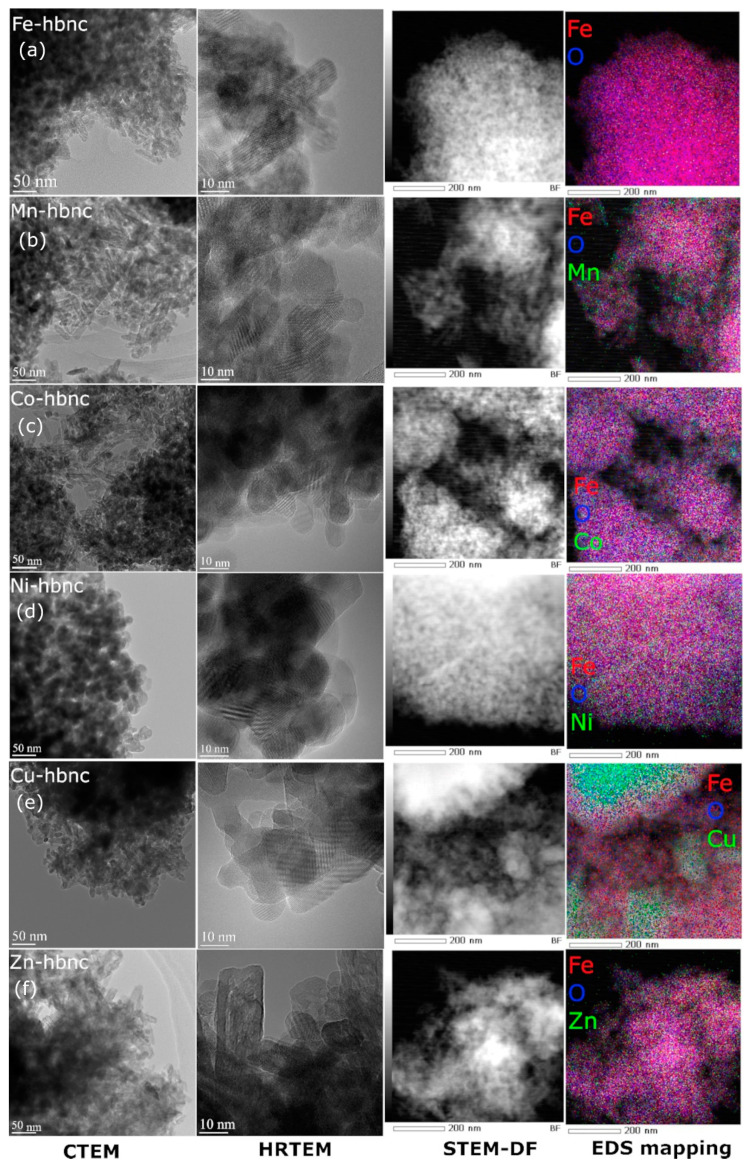
TEM results: CTEM, HRTEM, STEM, and EDS mappings for **Fe-hbnc** (**a**), **Mn-hbnc** (**b**), **Co-hbnc** (**c**), **Ni-hbnc** (**d**), Cu-**hbnc** (**e**), and **Zn-hbnc** (**f**).

The above XRD observations are confirmed by TEM results (Figure 4), where two types of nanoparticles can be observed, quasispherical (roughly 10 nm in size) and nanorod-like, with a width of roughly 10 nm and significantly higher length.

The undoped **Fe-hbnc** sample is composed of two subsystems of well-crystallized NPs: (i) quasi-spherical NPs with 10 nm size (α-Fe_2_O_3_) and (ii) nanorod-like NPs with a width of 10 nm and high length: width ratio (α-FeOOH). Lattice parameters from XRD do not differ from the reference ones. The corresponding Mössbauer spectra were described by a wide distribution of sextets on hyperfine fields and a supplementary sextet. The mean value of the HF in the distribution case is 51 T suggesting a defected α-Fe_2_O_3_ phase, whereas, in the case of the sextet, HF is 32 T, suggesting an α-FeOOH phase.

The **Mn-hbnc** and **Co-hbnc** samples have morphologies similar to the undoped ones. From the structural point of view, the α-FeOOH and α-Fe_2_O_3_ lattice planes are slightly compressed. The magnetic investigations suggest a higher degree of magnetic disordering than the undoped sample. The EDS mapping shows a uniform distribution of the dopant in the sample. It can be concluded that both components (α-FeOOH and α-Fe_2_O_3_) of the systems have been doped.

The **Ni-hbnc** sample comprises two subsystems of NPs having similar size and morphology: (i) ca. 20 nm α-Fe_2_O_3_ quasispherical NPs and (ii) ca. 40 nm γ-Fe_2_O_3_ quasispherical NPs. The XRD analysis shows a slightly c-compressed α-Fe_2_O_3_, while γ-Fe_2_O_3_ remained unaffected. The EDS mapping shows uniform dopant distribution. Mössbauer results summarized in Table 2 also show arguable magnetic disordering. It can be concluded that both components (α-Fe_2_O_3_ and γ-Fe_2_O_3_) may have been doped with Ni(II).

The **Cu-hbnc** sample comprises two subsystems of NPs having different sizes and morphologies: (i) ca. 10 nm α-Fe_2_O_3_ quasispherical NPs and (ii) elongated γ-Fe_2_O_3_ NPs. A ternary CuO phase is present due to copper segregation. Disregarding the CuO phase, which brings little or no contribution to the investigated effects, copper is uniformly distributed in the remainder of the sample.

While there is little change with the doping from the XRD point of view, the Mössbauer spectra, characterized by a distribution of sextets over hyperfine fields, suggest a high degree of magnetic disordering. A small paramagnetic phase imposed in the NORMOS is associated with a fraction of excellent paramagnetic NPs. It can be concluded that both components (α-Fe_2_O_3_ and γ-Fe_2_O_3_) of the systems have been doped with Cu(II).

The **Zn-hbnc** sample comprises two subsystems of NPs: (i) γ-Fe_2_O_3_ elongated NPs, and (ii) smaller α-FeOOH NPs. The uniform distribution of zinc observed in the elemental maps and the magnetic parameters observed in Table 2 suggest that both phases have been doped with Zn(II).

TEM observations are in agreement with the observations of Javed et al. (2019, 2020) on similar bimetallic compounds with Co(II) and Zn(II), where also the morphological features of the materials were investigated and exploited but for energy applications [38,39].

### 3.3. Textural Characterization of the Samples

**Figure 5:**
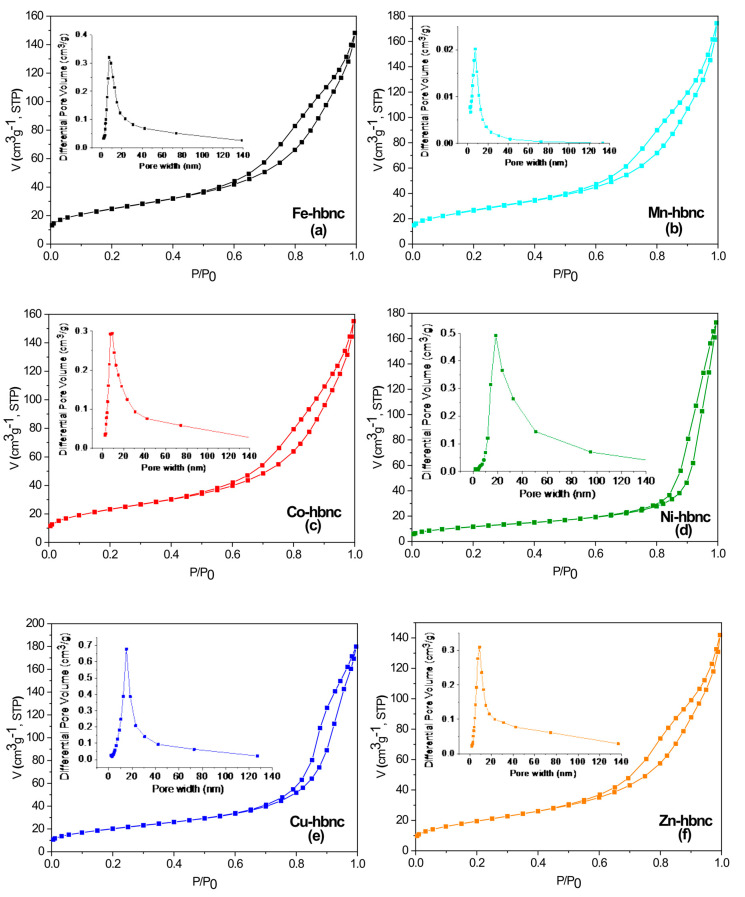
N_2_ adsorption–desorption isotherms and pore size distribution (inset) of the samples: **Fe−hbnc** (**a**), **Mn−hbnc** (**b**), **Co−hbnc (c)**, **Ni−hbnc** (**d**), **Cu−hbnc** (**e**), and **Zn−hbnc** (**f**).

Textural features of the samples were investigated by nitrogen adsorption–desorption analysis. Figure 5 shows the nitrogen sorption isotherms and the corresponding pore size distribution curves. All the isotherms are of type IV according to the IUPAC classification with hysteresis loops of H3 type, associated with capillary condensation phenomena in mesoporous structures [40].

As shown in Table 5, the specific surface areas decrease in the following order **Mn-hbnc** > **Fe-hbnc** > **Co-hbnc** > **Cu-hbnc** > **Zn-hbnc** > **Ni-hbnc**, from 94.8 (**Mn-hbnc**) to 42.3 m^2^g^−1^ (**Ni-hbnc**). Compared to the starting compounds, goethite and goethite-nanocomposites, a dramatic decrease (71–87%) of the surface area from 314–395 m^2^/g to 42–95 m^2^g^−1^ were observed as a result of thermal treatment. This behavior could be ascribed to the dehydroxylation of goethite/goethite-nanocomposites accompanied by the collapse of micropores and the formation of slit-shaped mesopores. The PSD curves reveal a monomodal distribution of pore sizes for all samples, except **Ni-hbnc**, with close average diameters ranging from 9.6 to 9.9 nm for the **Mn-**, **Fe-**, **Co-**, and **Zn-hbnc** samples and 13.27 nm for the **Cu-hbnc** one. In the case of **Ni-hbnc**, the pore size distribution is broader, extending over the whole domain associated with mesopores (2–50 nm). Total pore volumes of all samples are relatively close to each other, with values between 0.219 and 0.278 cm^3^g^−1^.

### 3.4. Characterization of Acid Base Properties

As seen from the results presented in Table 6, the **Mn-hbnc** sample has the highest number of acid sites, while the lowest number was determined for **Cu-hbnc**. Excepting **Co-** and **Zn-hbnc**, which had a balanced proportion of Lewis and Brønsted acid sites, for all the other samples, the proportion of Brønsted acid sites prevailed over the Lewis acid sites. The highest number of base sites was exhibited by **Cu-hbnc**, while the lowest one by **Zn-hbnc**. In terms of overall acid–base character expressed by the ratio between the total number of base sites and the total number of acid sites, all solids have a base character since the value of this ratio is higher than one, with **Cu-hbnc** being the most basic (ratio 2.8).

### 3.5. Catalytic Activity

Considering that the stoichiometric value needed for IC oxidation by hydrogen peroxide is 20 mol of H_2_O_2_ per mol of IC, the effect of varying the oxidant concentration in the reaction mixture was investigated over a wide range of H_2_O_2_/IC molar ratios. The effect of performing the tests without stirring was also investigated for one of the ratios.

The results of the catalytic tests performed with a simulated wastewater sample with an IC concentration of 0.03 mM are displayed in Table 7. In the absence of the catalyst, the dye removal extent (DR) was only due to the action of the oxidant, and it did not exceed 8%, even at a very high excess of H_2_O_2_ over the IC. In this case, the unreacted H_2_O_2_ at the end of the test was around 3 mgH_2_O_2_/L compared to 3.3 mg H_2_O_2_/L, the initial concentration determined by the analysis method described in the Supplementary Information. The absence of stirring leads to a decrease in DR by almost 20–30%, confirming that the adsorption of the reactants and desorption of the reaction products from the active catalytic sites are not spontaneous phenomena.

For all catalysts, the increase in H_2_O_2_/IC ratio was accompanied by a rise of DR percent. Excepting **Co-hbnc**, all the other modified hbnc samples showed better results than **Fe-hbnc**. Dye removal levels higher than 90% were obtained with **Ni-hbnc** and **Cu-hbnc** even at a slightly substoichiometric value of H_2_O_2_/IC. In these cases, the analyses of H_2_O_2_ after the reaction showed the total consumption of the oxidant.

These two catalysts also had an average dimension of pores larger than 13 nm and consequently allowed the access of the dye (molecular size > 10 nm, Supplementary Figure S3) to the active catalytic sites inside the pores. Since the dimensions of the pores are lower for all the other hbnc samples than the molecular size of the dye, the catalytic action took place only on the solid’s external surface, leading to lower DR values.

The results displayed in Table 8 show that the increase in the initial molar concentration of IC in the simulated wastewater at a molar ratio of H_2_O_2_/IC equal to 32.6 (a value slightly higher than the stoichiometric value) leads to the decrease in the DR% for all the catalysts besides **Cu-hbnc**, which exhibited DR% over 99%.

**Figure 6:**
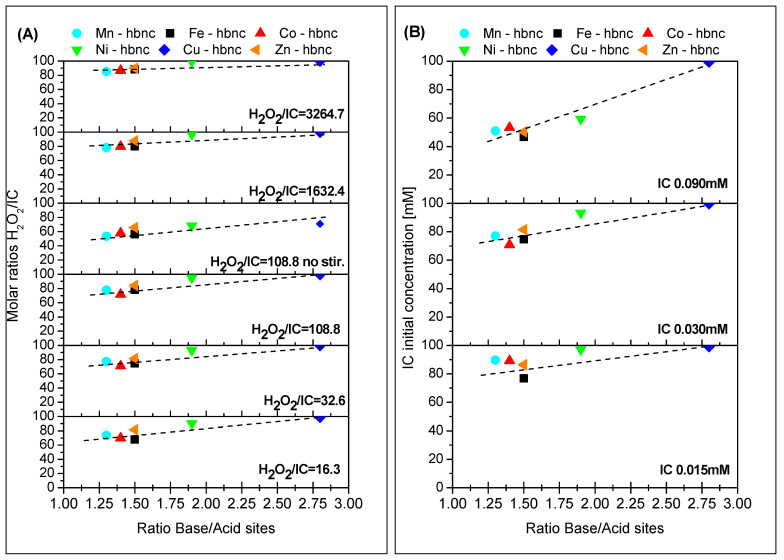
Linear correlation of the DR % and the ratio base/acid sites in the catalyst samples ((**A**)—IC initial concentration 0.03 mM, 25 °C, 2 h, 150 rpm, 1 wt% catalyst; (**B**)—molar ratio H_2_O_2_/IC = 32.6, 25 °C, 2 h, 150 rpm, 1 wt% catalyst).

The results in Table 7 and Table 8 correlate well with the catalyst samples’ base/acid site ratios (Table 6). The linear correlation is plotted in Figure 6.

**Figure 7:**
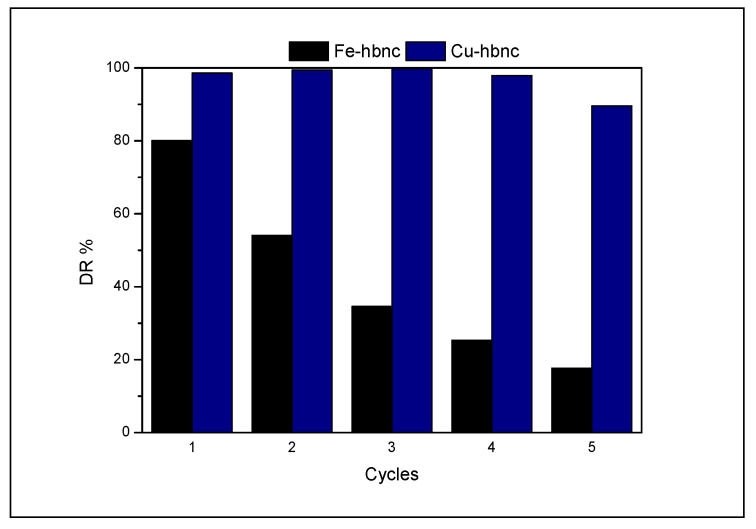
DR % during five reaction cycles on **Fe-hbnc** and **Cu-hbnc** (IC initial concentration 0.03 mM, molar ratio H_2_O_2_/IC = 32.6, 25 °C, 2 h, 150 rpm, 1 wt% catalyst).

The recycling tests performed for **Cu-hbnc** and **Fe-hbnc** catalysts plotted in Figure 7 show that **Cu-hbnc** preserves its catalytic activity with a DR higher than 90% after four recycles. The poorer results obtained on Fe-hbnc could be related to the leaching of iron species.

## 4. Conclusions

Some hematite-based nanocomposites were synthesized by controlled thermal decomposition of some goethite-based species previously reported [22]. These materials with different nano dimensions and shapes were characterized through data provided by Mössbauer spectroscopy, powder X-ray diffraction, and transmission electron microscopy. The pore volume and estimated diameter of the indigo carmine dye molecule evidenced that **Ni-hbnc** and **Cu-hbnc** samples provide access to the dye (molecular size > 10 nm) to the active catalytic sites inside the pores.

The catalytic test results for the oxidative degradation of indigo carmine dye from simulated wastewater with hydrogen peroxide evidenced that **Cu-hbnc** exhibits the highest activity. This performance can result from both textural properties (pore dimensions that allow the dye penetration) and the basic character of CuO. A good correlation between the DR% extent reached and the ratio of basic/acid sites of the catalysts has been established. It is worth mentioning that **Cu-hbnc** preserves its catalytic activity with a dye removal extent higher than 90% even after four recycles.

Compared to the other catalytic systems tested in the literature for the removal of IC dye, **Cu-hbnc** presents the advantage of being very active under mild conditions (atmospheric pressure, room temperature) without requiring a quartz photoreactor and a UV-light source like the previously reported TiO_2_ catalyst [26]. Besides that, the catalytic activity of **Cu-hbnc** is not inhibited by H_2_O_2_ oxidant and does not require an acidic pH as it is the case of MnO_2_ catalysts used for the removal of IC with persulfate at acid pH [27].

## Figures and Tables

**Table 1:** Absorption maxima (cm^−1^) in the IR spectra of samples.

Fe-hbnc	Mn-hbnc	Co-hbnc	Ni-hbnc	Cu-hbnc	Zn-hbnc	Assignment
3400 s, br	3390 s, br	3390 s, br	-	-	3410 s, br	ν(OH)
1630 w	1630 w	1630 w	-	-	1625 w	δ(OH)
1130 w	1120 w	1130 w	-	-	1110 w	δ(M-OH)
550 vs	550 vs	560 vs	575 vs	550 vs	550 vs	ν(Fe-O)
460 vs	460 vs	460 vs	460 vs	460 vs	450 vs	
405 s	405 s	405 s	405 s	405 s	405 s	

vs—very strong, s—strong, m—medium, w—weak, br—broad.

**Table 2:** Mössbauer hyperfine parameters (ISO-isomer shift (mm/s), QUA-quadrupolar shift (mm/s), HF-hyperfine field (T)) and relative area (%) of the Mössbauer spectral components. In the case of HF distribution, the two values provided for HF max correspond to the two most probable hyperfine fields.

	HF Distribution	Sextet/Doublet 1	Sextet/Doublet 2
**Fe-hbnc**	ISO = 0.19, QUA = −0.2, **average HF = 51 T**, Rel.area = 87.83%,	ISO = 0.27, QUA = −0.11, **HF = 32 T**, Rel.area = 12%	
	**88% Defected α-Fe_2_O_3_**	**12% α-FeOOH**	
**Mn-hbnc**	ISO = 0.2, QUA = −0.18, **average HF = 47 T**, Rel.area = 68%,	ISO = 0.29, QUA = −0.02, **HF = 19.6 T**, Rel.area = 32%	
	**68% Defected α-Fe_2_O_3_**	**32% α-FeOOH**	
**Co-hbnc**	ISO = 0.20, QUA = −0.2, **average HF = 45.68 T**, Rel.area = 89%,	ISO = 0.06, QUA = 0.39, **HF = 34**, Rel.area = 11%	
	**89% Defected α-Fe_2_O_3_**	**11% α-FeOOH**	
**Ni-hbnc**		ISO = 0.13, QUA = −0.04 **HF = 48 T**, Rel.area = 43%	ISO = 0.21, QUA = −0.17 **HF = 51 T**, Rel.area = 58%
		**43% γ-Fe_2_O_3_**	**58% α-Fe_2_O_3_**
**Cu-hbnc**	ISO = 0.14, QUA = −0.17, **average HF = 41.41 T**	Small paramagnetic phase (5%) with ISO = 0.22, QUA = 0.41	
	**Defected α-Fe_2_O_3_** **Defected γ-Fe_2_O_3_**		
**Zn-hbnc**	ISO = 0.21, QUA = −0.18, **average HF = 47 T**, Rel.area = 89%,	ISO = 0.39, QUA = −0.17, **HF = 28**, Rel.area = 11%	
	**89% Defected γ-Fe_2_O_3_**	**11% α-FeOOH**	

**Table 3:** Results of Rietveld analysis performed with MAUD software on XRD spectra.

Sample	Phase	a (Å)	b (Å)	c (Å)	Size (nm)	Quant (%)
**Fe-hbnc**	α-Fe_2_O_3_	5.030 ± 0.001	5.030 ± 0.001	13.763 ± 0.005	10.3 ± 0.1	83
	α-FeOOH	4.552 ± 0.006	10.067 ± 0.008	3.0125 ± 0.002	99.8 ± 41.6	17 ± 3
**Mn-hbnc**	α-Fe_2_O_3_	5.021 ± 0.0001	5.021 ± 0.0001	13.757 ± 0.003	11.9 ± 0.19	66
	α-FeOOH	4.619 ± 0.005	10.022 ± 0.009	2.989 ± 0.001	107 ± 62	34 ± 8
**Co-hbnc**	α-Fe_2_O_3_	5.025 ± 0.001	5.025 ± 0.001	13.74 ± 0.004	10.8 ± 0.2	81
	α-FeOOH	4.501 ± 0.002	10.152 ± 0.008	3.030 ± 0.001	94.5 ± 47.2	19 ± 2
**Ni-hbnc**	α-Fe_2_O_3_	5.032 ± 0.0001	5.032 ± 0.0001	13.74 ± 0.002	22.3 ± 0.19	33.3 ± 2
	γ-Fe_2_O_3_	8.324 ± 0.004	8.324 ± 0.004	25.024 ± 0.02	40.5 ± 2.8	66.7
**Cu-hbnc**	α-Fe_2_O_3_	5.034 ± 0.001	5.034 ± 0.001	13.71 ± 0.007	11.9 ± 0.14	72 ± 22
	γ-Fe_2_O_3_	8.335 ± 0.005	8.335 ± 0.005	25.051 ± 0.03	79 ± 31	12
	CuO	4.678 ± 0.002	3.419 ± 0.002	5.12 ± 0.004	81.8 ± 23.7	16 ± 6
**Zn-hbnc**	γ-Fe_2_O_3_	8.383 ± 0.004	8.383 ± 0.004	25.194 ± 0.03	272 ± 841	81.5
	α-FeOOH	4.604 ± 0.001	10.096 ± 0.010	2.915 ± 0.002	33.5 ± 3.7	18.5 ± 6

**Table 4:** Table of reference lattice parameters.

Phase (Reference)	a (Å)	b (Å)	c (Å)
α-Fe_2_O_3_	5.030	5.030	13.763
γ-Fe_2_O_3_	8.324	8.324	25.024
α-FeOOH	4.552	10.067	3.012

**Table 5:** Textural parameters of the samples.

Sample	S_BET_ (m^2^g^−1^)	Pore Volume (cm^3^g^−1^)	Average Pore Size (nm)
**Mn-hbnc**	94.8	0.269	9.84
**Fe-hbnc**	88.6	0.229	9.88
**Co-hbnc**	84.0	0.239	9.70
**Ni-hbnc**	42.3	0.267	21.00
**Cu-hbnc**	72.9	0.278	13.27
**Zn-hbnc**	71.8	0.219	9.67

**Table 6:** Acid–base properties of the samples.

Sample	Total Acid Sites	Acid Sites Distribution		Total Base Sites	Ratio Base/Acid Sites
	(mmol Py/g)	% Lewis	% Brønsted	mmol AA/g	
**Mn-hbnc**	0.352	22.1	77.9	0.459	1.3
**Fe-hbnc**	0.305	39.0	61.0	0.483	1.5
**Co-hbnc**	0.285	50.0	50.0	0.411	1.4
**Ni-hbnc**	0.207	31.4	68.6	0.393	1.9
**Cu-hbnc**	0.183	28.2	71.8	0.512	2.8
**Zn-hbnc**	0.250	51.5	48.5	0.375	1.5

**Table 7:** The dye removal extent (DR) after the oxidative degradation of IC at different molar ratios H_2_O_2_/IC (initial concentration of IC 0.03 mM, catalyst concentration 1 g/L, 150 rpm, 2 h at 25 °C).

Sample	Molar Ratios H_2_O_2_/IC					
	16.3	32.6	108.8	108.8 No Stirring	1632.4	3264.7
**Mn-hbnc**	73.6	77.1	77.6	53.5	78.0	85.2
**Fe-hbnc**	67.9	74.7	78.2	56.3	80.1	88.2
**Co-hbnc**	69.7	70.8	71.6	58.0	79.7	86.7
**Ni-hbnc**	90.3	93.2	95.1	68.3	97.2	99.5
**Cu-hbnc**	99.2	99.8	99.8	70.8	99.9	99.9
**Zn-hbnc**	81.5	81.5	84.3	65.8	87.9	89.8
**No catalyst**	4.2	4.2	5.1	2.5	5.8	7.3

**Table 8:** Oxidative degradation of IC at different molar ratios of the IC in water (H_2_O_2_/IC = 32.6; catalyst concentration 1 g/L, 150 rpm, 2 h at 25 °C).

Catalyst	IC Initial Concentration (mM)		
	0.015	0.030	0.090
**Mn-hbnc**	89.6	77.1	50.9
**Fe-hbnc**	76.8	74.7	46.7
**Co-hbnc**	89.2	70.8	53.3
**Ni-hbnc**	97.1	93.2	59.4
**Cu-hbnc**	99.2	99.8	99.4
**Zn-hbnc**	86.3	81.5	50.3

## Data Availability

Not applicable.

## Supplementary Materials

The following supporting information can be downloaded at: https://www.mdpi.com/article/10.3390/nano12142511/s1, Figure S1: Mössbauer spectra and associated NORMOS fits; Figure S2: X-ray diffractograms in Bragg–Brentano configuration (left) and the same diffraction pattern using d-spacing instead of diffraction angles (right); Figure S3: Optimized molecular structure of IC molecule was visualized by VESTA. References [41,42] were cited in Supplementary Materials.
