# Supplementary material for: Synthesis and Characterization of Hematite-Based Nanocomposites as Promising Catalysts for Indigo Carmine Oxidation
Source: Nanomaterials (Basel). 2022 Jul 21;12(14):2511. doi: 10.3390/nano12142511 (PMC9324592; doi:10.3390/nano12142511)
Supplement: Supplementary file 1 [file nanomaterials-12-02511-s001.zip › nanomaterials-1822541-supplementary.pdf]

Supplementary Material

Synthesis and Characterization of Hematite-Based Nanocomposites as Promising Catalysts for Indigo Carmine Oxidation

Andrei Cristian Kuncser [1], Arpad Mihai Rostas [1], Rodica Zavoianu [2], Octavian Dumitru Pavel [2,*], Ioana Dorina Vlaicu [1], Mihaela Badea [3], Daniela Cristina Culita [4], Alina Tirsoaga [5] and Rodica Olar [3,*]

1 Laboratory of Atomic Structures and Defects in Advanced Materials, National Institute of Materials Physics, 405A Atomiştilor Str., Ilfov, 077125 Măgurele, Romania; andrei.kuncser@infim.ro (A.C.K.); arpad.rostas@infim.ro (A.M.R.); ioana.vlaicu@infim.ro (I.D.V.)
2 Department of Organic Chemistry, Biochemistry and Catalysis, Faculty of Chemistry, University of Bucharest, 4–12 Regina Elisabeta Av., S3, 030018 Bucharest, Romania; rodica.zavoianu@chimie.unibuc.ro
3 Department of Inorganic Chemistry, Faculty of Chemistry, University of Bucharest, 90–92 Panduri Str., S5, 050663 Bucharest, Romania; mihaela.badea@chimie.unibuc.ro
4 Ilie Murgulescu Institute of Physical Chemistry, 202 Splaiul Independentei, 060021 Bucharest, Romania; dculita@icf.ro
5 Department of Physical Chemistry, Faculty of Chemistry, University of Bucharest, 4–12 Regina Elisabeta Av., S3, 030018 Bucharest, Romania; alina.tirsoaga@chimie.unibuc.ro
* Correspondence: octavian.pavel@chimie.unibuc.ro (O.D.P.); rodica.olar@chimie.unibuc.ro (R.O.)

The concentrations of H_2O_2 at the end of the catalytic tests were determined by the spectrophotometric methods 209 and 210 developed on Aqualytic spectrophotometer AL 800/SpectroDirect (Dortmund, Germany) using the specific reagent kits for the concentrations range of 0.01-0.5 mg/L and 0.03-1.5 mg/L H_2O_2.

Method 209 uses Hydrogen peroxide LR reagent tablets allowing the determination of concentrations in the range of 0.01-0.5 mg/L H_2O_2. Zero is determined with 50 mm cells filled with the water samples. After zero determination, the cell is emptied into a beaker, and the cell is dried completely. After rinsing the beaker with the water sample and empting it, a few drops of water should remain in the beaker and the Hydrogen peroxide LR reagent tablets has to be crushed inside the beaker using a clean stirring rod. After the tablet is crushed, 10 mL of water sample are added to dissolve the tablet. After dissolution the sample is transferred into the 50 mm cell of the spectrophotometer and after 2 minutes reaction time the measurement starts automatically.

Method 210 – uses Hydrogen peroxide LR reagent tablets allowing the determination of concentrations in the range of 0.03-1.5 mg/L H_2O_2. The procedure is similar to the above descried one, but instead of 50 mm cells the samples are poured in spectrophotometer vials (24 mm diameter).

The Mössbauer spectra as well as the associated NORMOS fits are presented in Figure S1.

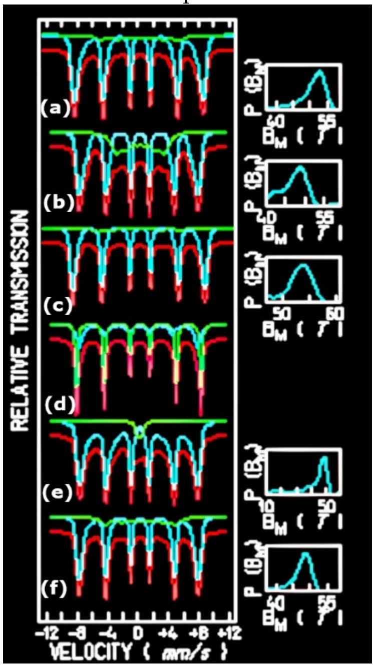

Figure S1. Mössbauer spectra and associated NORMOS fits.

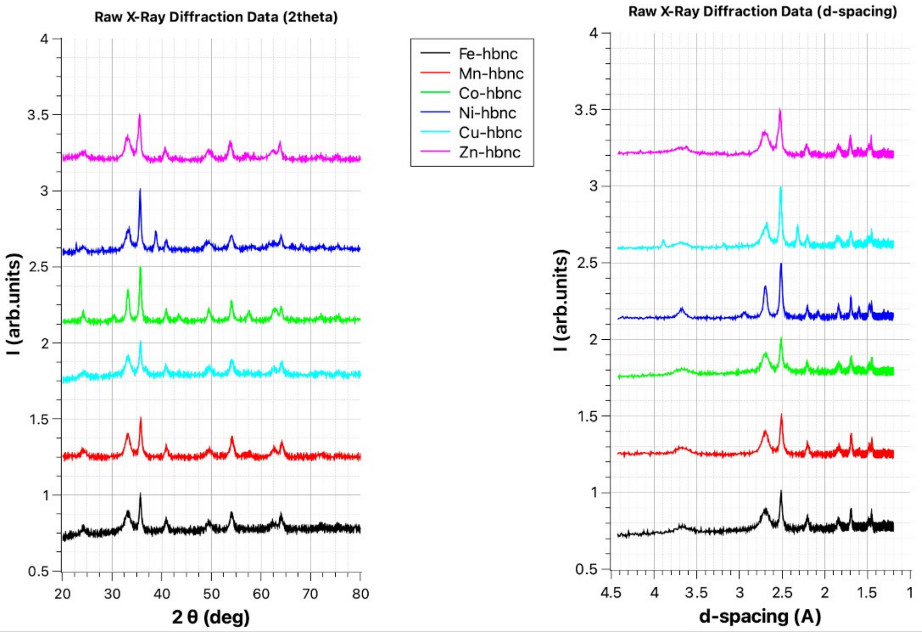

Figure S2. X-ray diffractograms in Bragg-Brentano configuration (left) and the same diffraction pattern using d-spacing instead of diffraction angles (right).

The optimized geometry of indigo carmine (IC) is shown in Figure S3. In order to estimate the diameter of the indigo carmine (IC) molecule an AM1 geometry optimization was performed with GAMESS 2021 R2 Patch 2 [41].

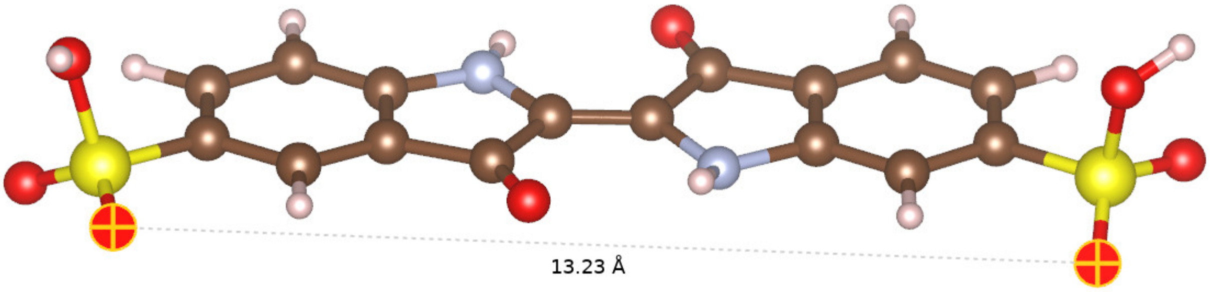

Figure S3. Optimized molecular structure of IC molecule visualized by VESTA [42].

The distance between the two S atoms is about 13.48 Å, while the distance between the two O atoms is about 13.23Å (Figure S2).

References

41. Barca, G.M.J.; Bertoni, C.; Carrington, L.; Datta, D.; De Silva, N.; Deustua, J.E.; Fedorov, D.G.; Gour, J.R.; Gunina, A.O.; Guidez, E.; Harville, T.; Irle, S.; Ivanic, J.; Kowalski, K.; Leang, S.S.; Li, H.; Li, W.; Lutz, J.J.; Magoulas, I.; Mato, J.; Mironov, V.; Nakata, H.; Pham, B.Q.; Piecuch, P.; Poole, D.; Pruitt, S.R.; Rendell, A.P.; Roskop, L.B.; Ruedenberg, K.; Sattasathuchana, T.; Schmidt, M.W.; Shen, J.; Slipchenko, L.; Sosonkina, M.; Sundriyal, V.; Tiwari, A.; Galvez Vallejo, J.L.; Westheimer, B.; Włoch, M.; Xu, P.; Zahariev, F.; Gordon, M.S. Recent developments in the general atomic and molecular electronic structure system. J. Chem. Phys. 2020, 152, 154102, https://doi.org/10.1063/5.0005188.
42. Momma, K.; Izumi, F. VESTA 3 for three-dimensional visualization of crystal, volumetric and morphology data. J. Appl. Crystallogr. 2011, 44, 1272-1276, https://doi.org/10.1107/S0021889811038970.
